# Incomplete datasets obscure associations between traits affecting dispersal ability and geographic range size of reef fishes in the Tropical Eastern Pacific

**DOI:** 10.1002/ece3.4734

**Published:** 2019-02-03

**Authors:** Adriana Alzate, Fons van der Plas, Fernando A. Zapata, Dries Bonte, Rampal S. Etienne

**Affiliations:** ^1^ Groningen Institute for Evolutionary Life Sciences University of Groningen Groningen The Netherlands; ^2^ Terrestrial Ecology Unit Ghent University Ghent Belgium; ^3^ German Centre for Integrative Biodiversity Research (iDiv) Halle‐Jena‐Leipzig Leipzig Germany; ^4^ Institute for Special Botany and Functional Biodiversity University of Leipzig Leipzig Germany; ^5^ Coral Reef Research Group, Department of Biology Universidad del Valle Cali Colombia

**Keywords:** adult mobility, aggregation behavior, body size, circadian activity, maximum linear distance, pelagic larval duration, spawning mode, tropical reef fishes

## Abstract

Dispersal is thought to be an important process determining range size, especially for species in highly spatially structured habitats, such as tropical reef fishes. Despite intensive research efforts, there is conflicting evidence about the role of dispersal in determining range size. We hypothesize that traits related to dispersal drive range sizes, but that complete and comprehensive datasets are essential for detecting relationships between species’ dispersal ability and range size. We investigate the roles of six traits affecting several stages of dispersal (adult mobility, spawning mode, pelagic larval duration (PLD), body size, aggregation behavior, and circadian activity), in explaining range size variation of reef fishes in the Tropical Eastern Pacific (TEP). All traits, except for PLD (148 species), had data for all 497 species in the region. Using a series of statistical models, we investigated which traits were associated with large range sizes, when analyzing all TEP species or only species with PLD data. Furthermore, using null models, we analyzed whether the PLD‐subset is representative of the regional species pool. Several traits affecting dispersal ability were strongly associated with range size, although these relationships could not be detected when using the PLD‐subset. Pelagic spawners (allowing for passive egg dispersal) had on average 56% larger range sizes than nonpelagic spawners. Species with medium or high adult mobility had on average a 25% or 33% larger range, respectively, than species with low mobility. Null models showed that the PLD‐subset was nonrepresentative of the regional species pool, explaining why model outcomes using the PLD‐subset differed from the ones based on the complete dataset. Our results show that in the TEP, traits affecting dispersal ability are important in explaining range size variation. Using a regionally complete dataset was crucial for detecting the theoretically expected, but so far empirically unresolved, relationship between dispersal and range size.

## 1 INTRODUCTION

A key question in macroecology and biogeography is why there is so much variation in the geographic range sizes of species (Gaston, 2003). Several explanations have been suggested for this large variation: environmental and physical constraints, species differences in niche breadth, population abundance, latitudinal gradients, species’ evolutionary age, body size, trophic level, colonization‐extinction dynamics, and dispersal ability (reviewed in Gaston, 2003), all of which have the potential to interactively cause variation in range size. However, empirical studies investigating these explanations are scarce and their conclusions are often conflicting (Gaston, 2003). This is especially the case for tropical reef fishes, for which a general consensus on the principal determinants of their range sizes remains elusive in spite of much research effort (Ruttenberg & Lester, 2015).

Dispersal is one of the most obvious processes related to range expansion (Sexton, McIntyre, Angert, & Rice, 2009). It influences demography, colonization dynamics, local adaptation, speciation, and extinction (Holt & Gomulkiewicz, 1997; Hubbell, 2001; MacArthur & Wilson, 1967). Because reef fishes are usually confined to discrete, often isolated habitats, dispersal is expected to be a particularly strong determinant of range sizes (Leis, 1991; Victor, 1991). However, evidence for the existence of a relationship between dispersal ability and geographical range size in reef fishes is mixed at best (reviewed in Lester & Ruttenberg, 2005; Ruttenberg & Lester, 2015). Pelagic larval duration (PLD), which is the time period fish larvae spend in the water column prior to settlement, thus a proxy of dispersal (the longer the PLD the greater the dispersal ability), has shown to correlate poorly with range sizes, which has led others to question the importance of dispersal (Lester, Ruttenberg, Gaines, & Kinlan, 2007; Mora et al, 2011) and to suggest that other life‐history traits are better predictors of range size (Luiz et al, 2013). Spawning mode (releasing either benthic or pelagic eggs) has been shown to be a good predictor of genetic structure in reef fishes (Riginos, Buckley, Blomberg, & Treml, 2014). However, despite the fact that genetic structure should reflect the level of connectivity among populations (i.e., level of dispersal; Clobert, Danchin, Dhondt, & Nichols, 2001), spawning mode has not been found to be a good predictor of range size so far (Luiz et al, 2013).

Although it seems obvious that dispersal should play a key role in determining the geographical ranges of species, the question is why the effects of dispersal have rarely been demonstrated conclusively. This can be due to (at least) two reasons. Firstly, most of the studies have investigated the effects on range size of only one dispersive life stage: the larval stage (but see Luiz et al, 2013). This is usually justified by the assumption that reef fishes significantly disperse only during this period, when they can be transported by ocean currents. The time that larvae spend in the ocean before settlement, pelagic larval duration (PLD), has been the main studied trait affecting dispersal ability (Victor, 1991). Nevertheless, dispersal consists of several stages (departure, transfer, and settlement) and occurs during different life stages (Bonte et al, 2012). Overall dispersal ability is the result of the combined effect of multiple traits, which could have evolved due to natural selection for high dispersal, or as an evolutionary by‐product (Burgess, Baskett, Grosberg, Morgan, & Strathmann, 2015). Dispersal in reef fishes also occurs during the egg and adult life stages (Addis, Patterson, Dance, & Ingram, 2013; Appeldoorn, Hensley, Shapiro, Kioroglou, & Sanderson, 1994; Kaunda‐Arara & Rose, 2004; Leis, 1978).

Although it is known that adults can move significant distances (Kaunda‐Arara & Rose, 2004), the precise effect of dispersal during the adult life stage on range size has to the best of our knowledge not yet been studied. Adult body size can indirectly be related to dispersal via its relationship to fecundity. Larger females tend to have a higher fecundity than smaller females, thus allowing more eggs or larvae to potentially reach distant, suitable habitat (Thresher, 1984; Wootton, 1992). By increasing propagule pressure during range expansion, the chances of survival during the transfer stage might be higher, which might positively affect large‐scale connectivity (Treml et al, 2012). Aggregation behavior and nocturnal activity have been suggested to be related to the settlement stage of dispersal by decreasing predation risk, which increases colonization success (Luiz et al, 2013). Thus, various traits are directly or indirectly related to dispersal, and focusing on only one or very few of them might preclude the detection of links between dispersal ability and range sizes.

A second possible reason why it has been difficult to relate dispersal ability to range size is more methodological. Studies examining the range size–dispersal ability relationship in reef fishes have used only a subset of species (e.g., groups of species with known information on the trait of interest). The reason for this is that when investigating relationships between PLD and range size, scientists are limited by the relatively scarce availability of data. For instance, in the Tropical Eastern Pacific (TEP), this trait has been estimated for only 30% of the species. Studying a subset of species to test an ecological hypothesis is a common, more or less accepted practice in macroecology (Blackburn & Gaston, 1998). Nevertheless, an implicit assumption behind the use of species subsets is that this subset is representative of the total species pool, which may not always be the case. As pointed out by Blackburn and Gaston (1998), missing species do not only add noise to the macroecological patterns but might also distort them. Currently, we do not know the consequences of using only the subset of species for which PLD has been estimated when studying range sizes in tropical reef fishes.

Here, we tried to overcome some of the difficulties mentioned above by firstly investigating the effect of six dispersal‐related traits (adult mobility, spawning mode, PLD, circadian activity, aggregation behavior, and body size) on range size, using only the subset of species for which PLD data is available (PLD‐subset, 148 species). Secondly, we used null models to investigate whether the trait distribution for species in the PLD‐subset is representative of the regional species pool. Then, we analyzed the complete dataset of reef fishes in the TEP (using five traits instead of six because of the need to exclude PLD data) to test whether (and if so, why) the results regarding the drivers of range sizes are different from those obtained when using only the PLD‐subset. Finally, we investigated how using an incomplete set of species may affect statistical model outcomes by analyzing 1,000 random subsets with the same number of species as the PLD‐subset. We focus our study on the distribution of tropical reef fishes present in the TEP, which is a well‐defined region with relatively clear limits, relatively isolated from other marine regions (such as from the Caribbean by the Panama Isthmus and from the Indo‐Pacific by the large span of open ocean known as the East Pacific Barrier). The TEP marine fish fauna is well known, and information on geographic distribution and other species traits is available (Froese & Pauly, 2011; Robertson & Allen, 2016).

## 2 METHODS

### 2.1 Range size data

Data on geographic distribution of reef fish species were obtained from the Shorefishes of the Tropical Eastern Pacific (SFTEP), Online Information System (Robertson & Allen, 2016), and the IOBIS database (to complete the distributions of species that are not endemic to the TEP). We restricted our study to the distribution of tropical reef‐associated bony fishes in the TEP region (sensu Robertson & Allen, 2016). We included only tropical species, whose main distribution occurs in the Pacific Ocean. For a total of 497 species, we calculated range size using the geographical coordinates of all records reported in the region. Range size was measured as the maximum linear distance (in kilometers) between any two points where a species has been recorded (Gaston, 1994). Range size was calculated using the function “geodist” from the R package “gmt” (Magnusson, 2014).

### 2.2 Predictors of range size

We collated information on several species traits potentially affecting dispersal from the literature and online databases: body size, adult mobility, spawning mode, PLD, circadian activity, and aggregation behavior. All of these factors have been suggested as possible drivers of range size in reef fishes (e.g., Lester et al., 2007; Luiz et al, 2013; Ruttenberg & Lester, 2015).

Body size, the maximum recorded total length for each species, was obtained from Fish Base (Froese & Pauly, 2011) and Shore Fishes of the Tropical Eastern Pacific online information system—SFTEP (Robertson & Allen, 2016). Body size is related to many other life‐history traits, also to habitat specialization and predation risk, which consequently could affect range size (Calder, 1984; Peters, 1983). Body size is positively related to fecundity (Thresher, 1984; Wootton, 1992; Zapata, 1990), increasing propagule pressure during range expansion and probably influencing large‐scale connectivity (Treml et al, 2012). It is also positively related to adult mobility (Barlow, 1981) and home range size (Nash, Welsh, Graham, & Bellwood, 2015; Peters, 1983; Welsh & Bellwood, 2014), thereby potentially leading to larger range sizes (Gaston, 2003; Gaston & Blackburn, 1996).

Adult mobility was classified as low, medium, or high following Floeter, Ferreira, Dominici‐Arosemena, and Zalmon (2004). Information for each species was collated from several studies (Appendix S1). Data on spawning mode were obtained from SFTEP (Robertson & Allen, 2016). Species were classified as pelagic or nonpelagic spawners (including species with benthic eggs, mouthbrooding and live birth). Pelagic spawners release their eggs in the water column, which are passively transported by water currents until the larvae hatch and are able to swim actively (Leis et al, 2013; Stobutzki, 1997). Thus, spawning mode gives an indication of dispersal in both the egg and larval stage (Leis, 2006; Leis et al, 2013). Data on pelagic larval duration (PLD) was obtained from the literature (Appendix S1). We supplemented these data with viviparous species, for which PLD = 0. Adult mobility, spawning mode, and PLD are all traits related to (but not actual measures of) dispersal ability that act at different life‐history stages.

Data on circadian activity were obtained from Fish Base (Froese & Pauly, 2011), SFTEP (Robertson & Allen, 2016), as well as several other studies (Appendix S1). Species were classified as diurnal, crepuscular, or nocturnal. Data on aggregation behavior were obtained from Fish Base (Froese & Pauly, 2011), SFTEP (Robertson & Allen, 2016), and several other studies (Appendix S1). Species were classified as nonaggregative (species that never form any aggregation), temporarily aggregative (species that at some point in their lives form spawning or feeding aggregations), and aggregative (species that form aggregations or schools). Information on trophic level was collated from Fish Base (Froese & Pauly, 2011). Schooling (a form of aggregation) and nocturnal activity (a type of circadian activity) have been previously found to be good predictors of range size for tropical reef fishes (Luiz et al, 2013). They are suggested to reduce predation risk and increase the chances of survival and establishment after settlement (Luiz et al, 2013).

### 2.3 Data analysis

#### 2.3.1 Analysis of the PLD‐subset

We used general linear mixed models (LMMs) to study which factors explain variation in range size for reef‐associated fish species in the TEP. We focused first on the species subset (148 species) for which PLD data is available. The model included six fixed factors, which are the species traits also described above: body size, spawning mode (pelagic or nonpelagic spawners), adult mobility (low, medium, or high), PLD, circadian activity (diurnal, crepuscular, or nocturnal), and aggregation behavior (temporarily aggregative, aggregative, nonaggregative). In addition, we controlled for possible phylogenetic effects (phylogenetic conservatism of range size) by including Genus, Family, and Order as nested random factors in the model. With an additional random factor (ocean basin), we controlled for the origin of the fauna: We distinguished between species that are endemic (TEP endemic) and species that are nonendemic to the TEP (e.g., transpacifics). Using ocean basin as a random factor follows similar approaches used in previous studies (Luiz et al., 2013). We included this factor because transpacific species that reached the TEP have disproportionally larger range sizes than TEP endemics, which is due to differences in the largest distance between any two points within each ocean basin (~19,000 km vs. ~ 6,000 km).

We included a random factor that controls for the particular spatial structure of the TEP (a long continuous continental coastline + scattered oceanic islands), which imposes two different possible maximum range sizes (one for species that live on the coastline [~6,000 km] and one for the species living only on oceanic islands [~3,500 km]). The maximum linear distance between the northern and southern tips of the TEP is much longer than most of the island‐mainland distances or island‐island distances, potentially allowing species that range widely over continuous habitat to have broader geographic ranges than species that cross the gap between the islands and the mainland.

Predictors were tested for multicollinearity using the R code “HighstatLib.r” from Zuur, Ieno, and Elphick (2010). Variance inflation factors (VIF) of all variables were below 2.5, which is considered adequate for ensuring that variables are not collinear (Zuur et al., 2010). Range size data were logit‐transformed to meet linear mixed model assumptions (Appendix S2).

The model was fitted using the “lmer” function from the R package “lme4” (Bates et al., 2014). We standardized the explanatory variables, in order to compare the effect sizes of the different predictors, using the function “standardize” from the R package “arm” (Gelman et al., 2009). We performed model selection using the function “dredge” from the R package “MuMIn” (Barton, 2013). To ensure robust model estimates, we used model averaging across all models (with an Akaike weight larger than 0.001) nested within the global model (Grueber, Nakagawa, Laws, & Jamieson, 2011). Model averaging was done using the function “model.avg” from the R package “MuMIn” (Barton, 2013), which runs all possible models nested within the global model, calculates effect sizes of each predictor of each model, and calculates average effect sizes across models, weighed by Akaike weights.

We then used null models to test whether the PLD‐subset is representative for the regional species pool, in terms of taxonomical, ecological, and life‐history traits. We compared trait averages of 10,000 random subsets extracted from the complete dataset, each with 148 species (the same number of species as those with available PLD data), with the traits of the PLD‐subset by calculating the difference in average trait values between the random and PLD‐subset. Random subsets required that all levels per factor contained at least one data point (species). If average trait values of the PLD‐subset were higher or lower than average trait values in 9,750 out of 10,000 random subsets, the difference was considered significant (two‐sided test; *α* = 0.05).

#### 2.3.2 Analysis of the complete dataset and random subsets

we repeated the general linear mixed models (LMMs) described above for the PLD data subset, but this time including all reef‐associated fish species in the TEP. The only difference was that we could not include PLD as a predictor, due to data incompleteness.

As a sensitivity analysis, we repeated this model 1,000 times, but using random data subsets as described above. This way, we could investigate whether the analysis of random subsets of species, including species without PLD measurements, and without PLD as a predictor, yields the same results as the model for the PLD‐subset. Random subsets required the presence of at least one data point (species) per level for each factor. The fitting and model selection procedures were the same as used for the PLD‐subset.

## 3 RESULTS

### 3.1 Drivers of range size in the PLD‐subset

**Figure 1:**
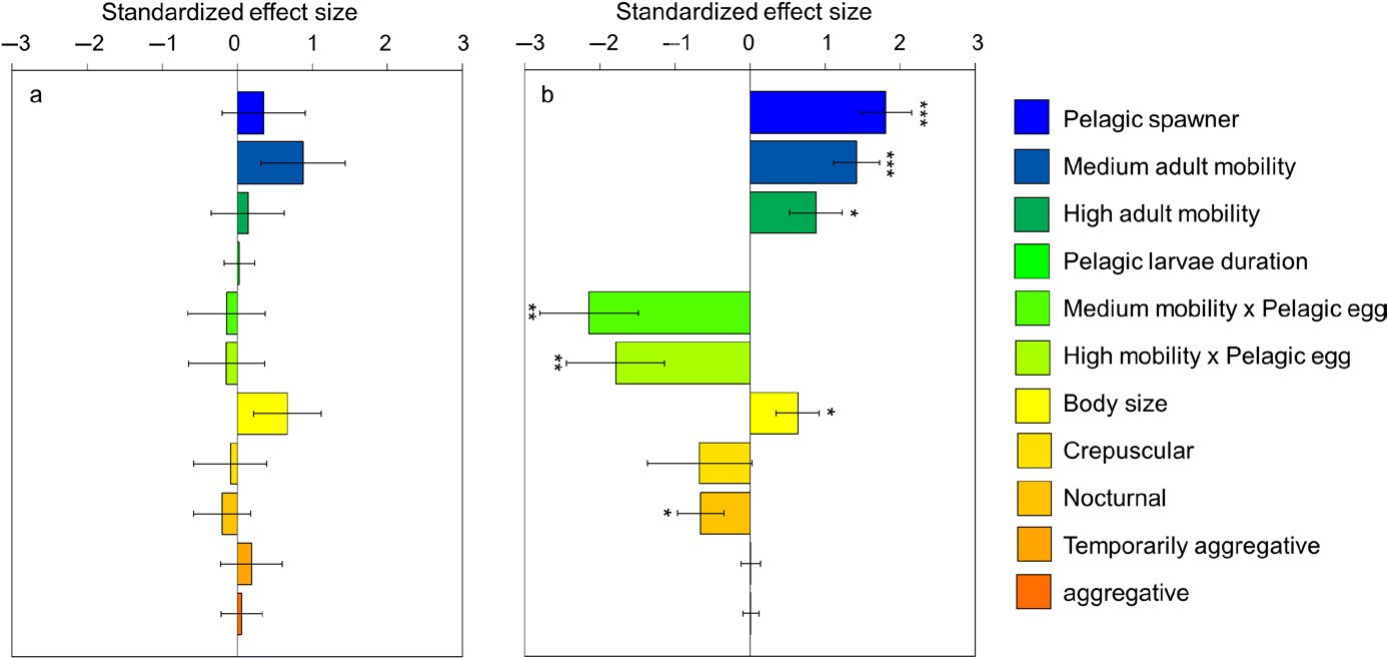
Standardized effect sizes of different predictors of range size for (a) the PLD‐subset and (b) the complete dataset. Standard errors and significance levels (* <0.05, ** <0.001, *** <0.0001) are shown. Reference levels: nonpelagic eggs, low adult mobility, nonaggregative, and diurnal

**Figure 2:**
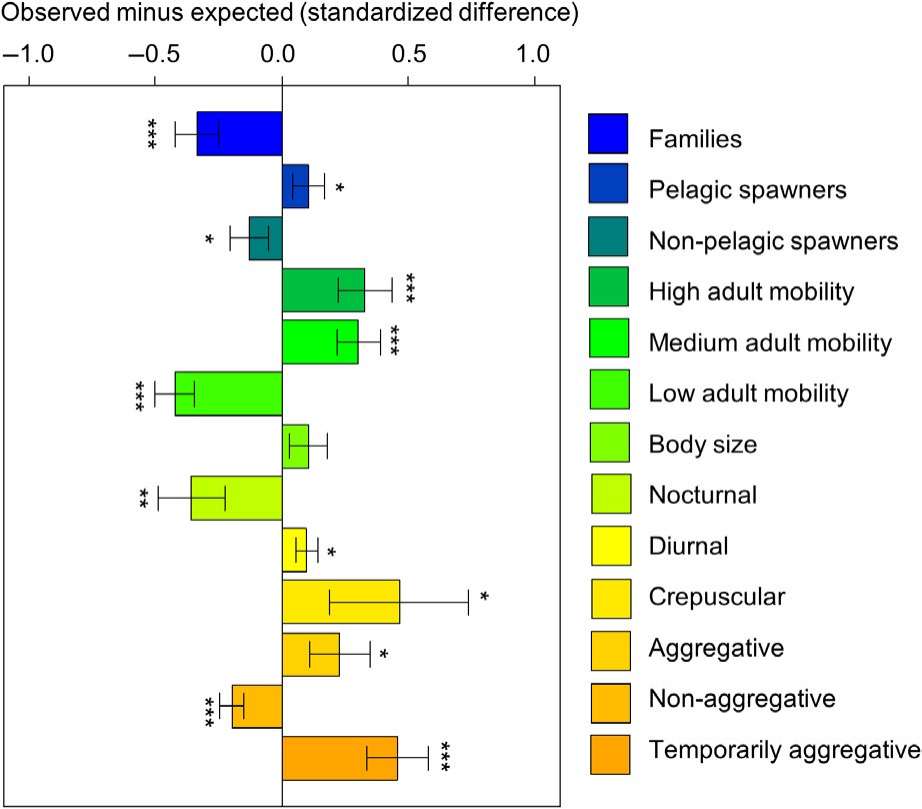
Standardized difference between the average trait value of 10,000 random species subsets and the PLD‐subset. Both the random and the PLD‐subset contained the same number of species (148). Positive values mean that the random subsets show higher values than the PLD‐subset and vice versa for negative values. 95% confidence intervals and significance levels (* <0.05, ** <0.001, *** <0.0001) are shown

When analyzing the drivers of range sizes using the PLD‐subset, we found that none of the studied traits had a significant effect on range size (Figure 1a, Table [Link] in Appendix S3). Null models indicated, however, that most trait values from the PLD‐subset significantly differed from traits values obtained from randomly drawn species subsets (Figure 2). The PLD‐subset contained, on average, a lower number of families (indicating a narrower phylogenetic breadth), a lower proportion of nonpelagic spawners, and a lower proportion of nonaggregative, nocturnal, and low mobility species than random subsets. The PLD‐subset contained a higher proportion of pelagic spawners, and a higher proportion of aggregative, temporarily aggregative, diurnal, crepuscular, medium, and high mobility species than random subsets. The only trait that did not significantly differ between the PLD‐subset and randomly drawn species subsets was adult body size.

### 3.2 Predictors of range size in the complete TEP data and random data subsets

**Figure 3:**
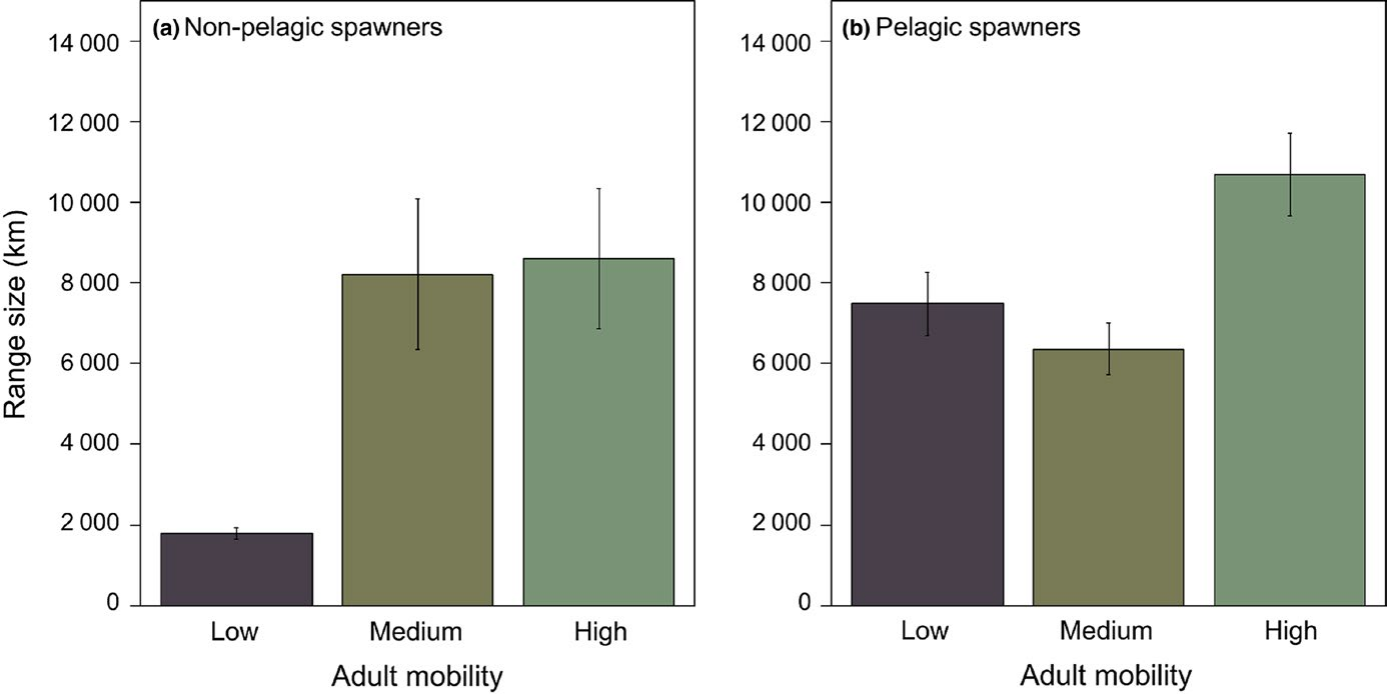
(a) range size significantly increases with adult mobility for species of nonpelagic spawners. (b) Range size is generally larger for pelagic spawners than for nonpelagic spawners, but does not significantly increase with adult mobility within the group of pelagic spawners. Standard errors are shown

An analysis of the complete dataset, including all tropical species in the TEP, but excluding PLD from the model (because there is not information for all species), revealed that the range size of reef fishes in the TEP is positively associated with several traits directly or indirectly related to dispersal ability (Figure 1b; Table 1 in Appendix S3). Spawning mode and adult mobility significantly interact to affect species range size (Figure 3 a,b, Table 1 in Appendix S3). While species that are pelagic spawners have larger ranges than nonpelagic spawners (by on average 5,070 km), the effect of adult mobility depends on the type of spawning mode (Figures 1 and 3 a,b). We found a strong positive relationship between adult mobility and range size for nonpelagic spawners, but not for pelagic spawners. Among nonpelagic spawners, species with high adult mobility have on average a range 6,829 km greater than that of species with low adult mobility. Body size and circadian behavior are also correlated with range size. Whilst body size has a positive effect on range size, nocturnal species attained significantly smaller ranges than diurnal species (Figure 1b, Table 1 in Appendix S3).

**Figure 4:**
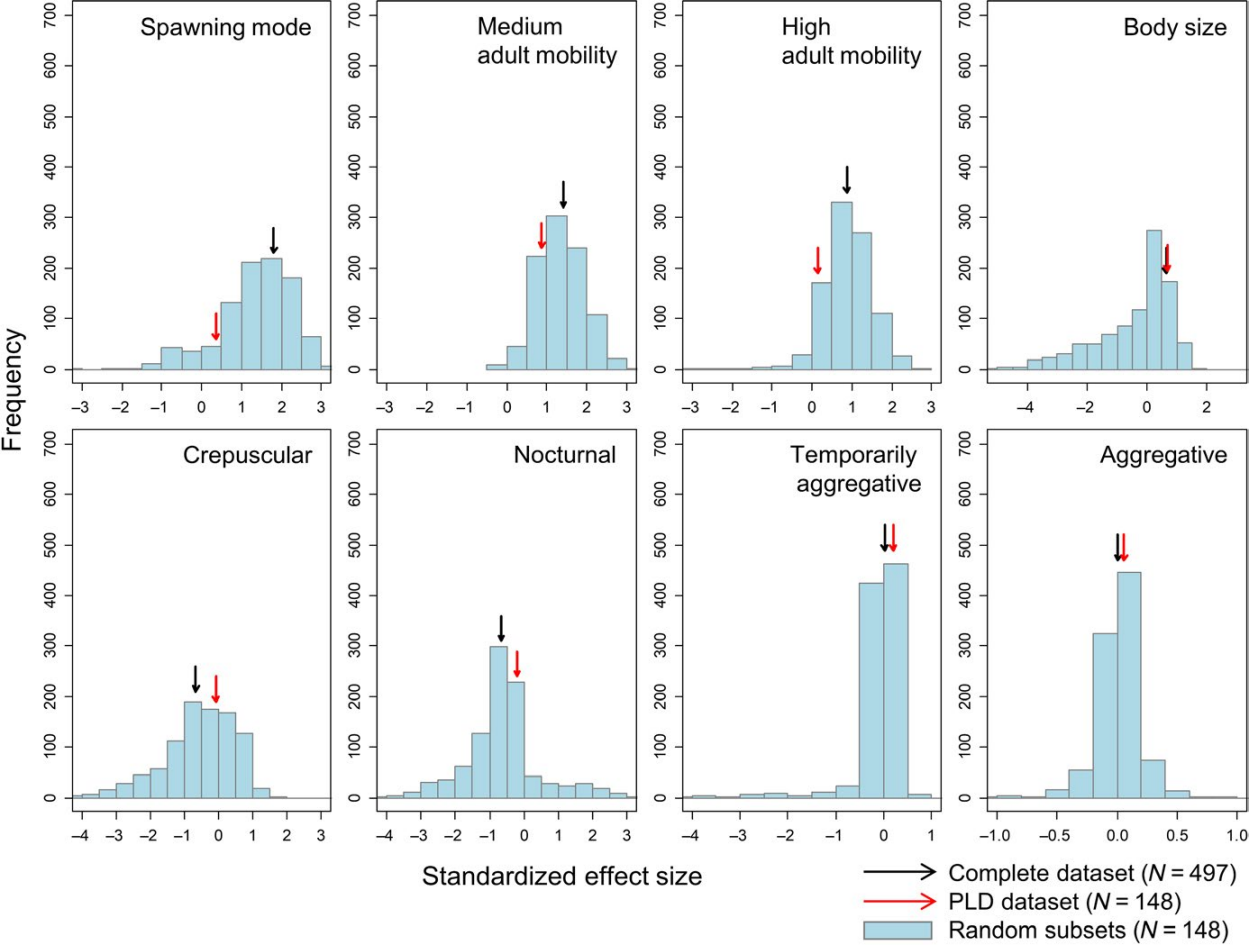
Frequency distributions of effect sizes of model coefficients for 1,000 linear mixed model analyses of equal number of random species subsets. The black and red arrows show the effect size values for the complete dataset and the PLD‐subset, respectively

**Figure 5:**
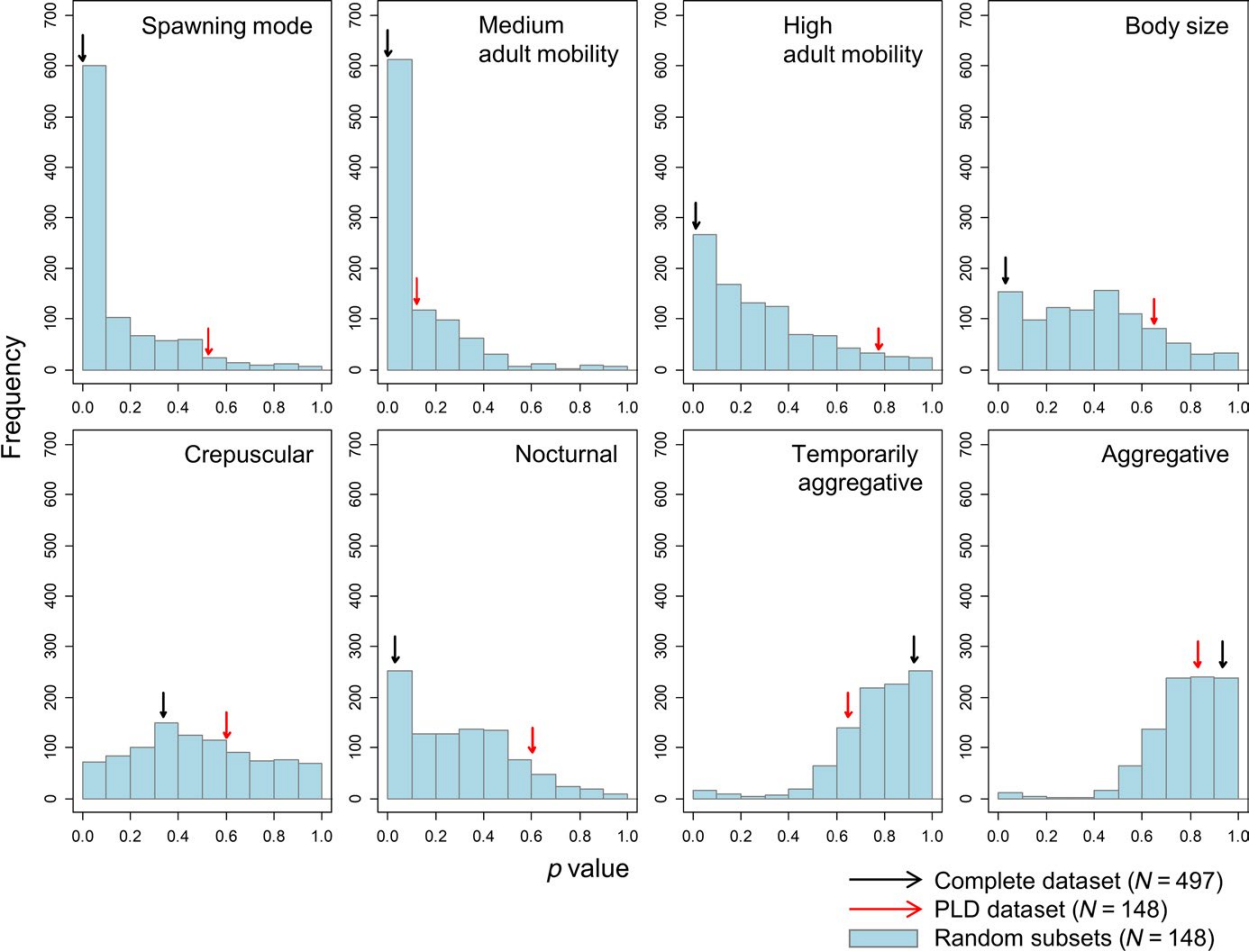
Frequency distributions for the significance of model coefficients for 1,000 random species subsets. The black and red arrows show the significance values for the complete dataset and the PLD‐subset, respectively

We performed the same statistical analysis for 1,000 random species subsets (with the same number of species as the PLD‐subset). The effect sizes and significance levels (modal values) estimated from random species subsets were generally very similar to the ones estimated using the complete dataset (Figure 4). In contrast, effect sizes and significance levels of both the complete dataset and those of random subsets substantially differed from those of the PLD‐subset for spawning mode and adult mobility: In the PLD‐subset, positive effects of pelagic spawning mode and medium/high mobility on range size were underestimated (Figure 4). Furthermore, in the majority of cases, the *P* values by which spawning mode (88.9% of cases), medium (64.5% of cases), and high (89.7% of cases) adult mobility were related to range size were lower (i.e., more significant) in models based on random species subsets than in the PLD‐subset, even though sample sizes (and therefore statistical power) were equal (Figure 5).

## 4 DISCUSSION

In spite of the obvious theoretical importance of dispersal as a potential determinant of range size (especially for organisms living in highly spatially clustered habitats), supporting empirical evidence has so far been surprisingly limited in both terrestrial (Dennis, Donato, Sparks, & Pollard, 2000; Edwards & Westoby, 1996; Gaston & Blackburn, 2003; Gutierrez & Menendez, 1997; Juliano, 1983; Kavanaugh, 1985; Malmqvist, 2000; McCauley, Davis, Werner, & Robeson, 2014) and marine organisms (Bonhomme & Planes, 2000; Brothers & Thresher, 1985; Hansen, 1980; Jablonski & Lutz, 1983; Jones, Caley, & Munday, 2002; Lester & Ruttenberg, 2005; Lester et al., 2007; Luiz et al, 2013; Mora et al, 2011; Mora, Chittaro, Sale, Kritzer, & Ludsin, 2003; Nanninga & Manica, 2018; Ruttenberg & Lester, 2015; Thresher & Brothers, 1985; Thresher, Colin, & Bell, 1989; Victor & Wellington, 2000; Wellington & Victor, 1989; Zapata & Herrón, 2002). Our study illustrates several reasons why it is difficult to find a definite answer to the question of whether or not dispersal is an important determinant of species range sizes. The first reason is the use of species subsets to tackle macroecological questions. We showed that when using the subset of species for which PLD data is available, none of the studied species traits significantly affected range size in the TEP. In contrast, when the dataset includes all species (but not necessarily all traits), several traits affecting dispersal ability are positively correlated with species range sizes, at least in the TEP. A possible explanation is that the use of small data subsets does not allow to capture the true nature of the relationship between traits affecting dispersal ability and range size simply because of lack of statistical power. We tested this by analyzing random subsets of the TEP species pool and comparing the model outcomes with those based on the complete dataset and the PLD‐subset. In contrast to analyses based on the PLD‐subset, but in agreement with the analysis based on the complete dataset, most models based on random species subsets indicated that both spawning mode and adult mobility are significantly positively correlated with range size. Thus, the lack of relationship between range size and traits related to dispersal ability is not likely caused by the used of smaller datasets per se, but by the particular nature of the PLD‐subset, which consists of a highly nonrandom set of species.

The fact that PLD has only been estimated for 30% of the reef‐associated species in the TEP (497 in total) and that 32% of these species come from only three families (Labridae =13.6%, Pomacentridae =10.7%, and Serranidae =7.9%) could have been a reason for why we did not detect any signal in the analysis of the PLD species subset. We showed, using null models, that the PLD‐subset is not a representative sample from the regional species pool: 12 out of 13 characteristics that we compared differed significantly between the PLD‐subset and random species subsets. Therefore, the PLD‐subset for the TEP is biased in terms of taxonomical breadth and species traits. It contains a smaller number of families, a lower proportion of nocturnal, nonpelagic and low mobile species and a higher proportion of diurnal, aggregative and more dispersive species (pelagic spawners with high/medium adult mobility) than expected based on random species subsampling. Potential problems related to the used of biased datasets have previously been discussed verbally (Blackburn & Gaston, 1998). Our study quantitatively shows that these problems are far from trivial and that biased datasets can have severe implications for the outcomes and conclusions drawn from a study. A similar example of such an effect is shown in a study examining how species traits influence extinction risk in mammals, where a seemingly strong relationship between body mass and extinction risk (larger mammals being more vulnerable) is actually driven by the biased availability of data, where large (usually rare) mammals have been generally better studied than small ones (Gonzalez‐Suarez, Lucas, & Revilla, 2012). Therefore, general conclusions based on the analysis using the PLD‐subset should be taken with extreme caution, because the nonrandom sample from the regional species pool may bias model estimates (Little & Rubin, 2002; Nakagawa & Freckleton, 2008).

A second reason why we found, in contrast to many studies, a clear relationship between dispersal and range size, is that most studies have focused on single or few dispersal‐related traits, thereby (implicitly) denying the fact that dispersal is more complex including traits related to departure, transfer, and settlement decisions. The inclusion of other traits likely to affect dispersal ability (e.g., spawning mode, body size, adult mobility) revealed that several of these traits, some of which are typically not studied, are strong predictors of range sizes. Our analyses of the complete dataset, as well as those based on random subsets, showed that several traits related to different stages of dispersal (spawning mode, body size, and adult mobility) correlate positively with range size in the TEP. Species that are pelagic spawners that are more mobile during their adult stage and that attain larger body sizes have larger ranges than nonpelagic spawner, less mobile, and smaller‐bodied species. The effect of adult mobility on range size depends on the type of spawning mode. While adult mobility does not affect range size of pelagic spawners, it does significantly affect the range size of nonpelagic spawners. In this study, we could not detect significant relationships between PLD and range sizes. Similarly, another study has shown that larval swimming capacity is a better proxy of dispersal than PLD per se (Nanninga & Manica, 2018). Nevertheless, the importance of PLD for range size cannot be completely discarded until more complete information on this trait is available for a larger number of species.

Pelagic spawners are able to attain larger ranges than nonpelagic spawners because pelagic eggs spend some time in the water column, which allows for passive dispersal without experiencing the energetic costs that larvae do (Leis et al, 2013; Stobutzki, 1997). Larvae of nonpelagic spawners hatch at a more developed stage and are more developed at hatching. Thus, have better control of their dispersal than those of pelagic spawners (Leis et al, 2013; Wootton, 1992). More mature larvae become more active swimmers and are less likely to passively disperse for long distances through ocean currents (Leis, 1991, 2006; Leis et al, 2013; Munday & Jones, 1998; Stobutzki & Bellwood, 1997). Therefore, passive dispersal is expected to be the predominant dispersal mode at least at the beginning of the larval stage for pelagic spawners, whereas active dispersal is likely to be predominant for nonpelagic spawners earlier in their larval life.

The positive effect of body size on range size is likely to be more indirect, as body size can affect fecundity, dispersal during the egg and larval stage, survival, and adult mobility. Larger females usually have higher fecundities, which means more propagules and therefore a higher probability that at least some of those propagules (larvae) will survive and colonize distant localities (Thresher, 1984; Wootton, 1992; Beldade et al, 2012). An increase in propagule pressure during range expansion can have consequences for large‐scale connectivity (Treml et al, 2012). In addition, larger species tend to produce smaller eggs which are more likely to be dispersed passively (Thresher, 1984). Body size is positively related to adult mobility (Barlow, 1981) and home range size (Welsh & Bellwood, 2014), thereby potentially leading to larger range sizes (Gaston, 2003; Gaston & Blackburn, 1996). In contrast to another study based on all ocean basins (Luiz et al, 2013), our study did not find relationships between range size and aggregation behavior. This trait has been treated as proxies for predation avoidance and subsequent successful colonization of new habitat patches (Luiz et al, 2013), but our study cannot provide evidence for the importance of this factor in explaining range sizes in the TEP. We also found a negative relationship between nocturnality and range sizes. This was an unexpected result and in contrast to previous findings (Luiz et al, 2013).

Another possible reason why our results contrast with other studies that did not find strong links between traits that affect dispersal ability and range size is that mechanisms underlying range expansion in the TEP might differ from mechanisms that predominate in other regions. First, all oceans basins differ in size, shape, geographical barriers, habitat availability, and configuration, which can influence the maximum range that species can attain (Ruttenberg & Lester, 2015). In the TEP, the reef habitat available for reef fishes is mostly distributed along the coast in a semicontinuous manner (except for two wide stretches of sand that act as dispersal barriers or filters) and around the few oceanic islands. Such extrinsic factors could not only influence range size, but also which and how species traits influence range expansion. Certainly, other factors, not studied in detail here, such as species age and geological history (e.g., habitat suitability and isolation; Pellissier et al, 2014; Ruttenberg & Lester, 2015) also might drive variation in range sizes, and it is likely that these factors interact with dispersal. Hence, future studies investigating these factors simultaneously, using (regionally) complete datasets, might provide even greater understanding in the distribution of range sizes.

In summary, we showed that several traits presumed to affect dispersal ability are important determinants of range size and that using several of these traits and a regionally complete dataset, rather than a subset of the species in a region, was crucial for reaching these conclusions. Our study serves as a warning about the crucial importance of choosing an adequate dataset to study macroecological patterns. We demonstrated that the use of a species subset, which is not a random sample from the regional species pool (the PLD‐subset), can produce very different results than the complete dataset or subsets based on random samples.

## AUTHORS’ CONTRIBUTION

AA developed the research idea and collated the data. AA and FvdP performed the statistical analyses. AA wrote the first draft of the manuscript. AA, FvdP, FAZ, DB, and RSE contributed with discussions and comments on the manuscript.

## Data Availability

Data on range size and traits of reef fishes used in this study are available on the Dryad digital repository https://doi.org/10.5061/dryad.ns5m4kg.
