# Supplementary material for: Incomplete datasets obscure associations between traits affecting dispersal ability and geographic range size of reef fishes in the Tropical Eastern Pacific
Source: Ecol Evol. 2019 Feb 3;9(4):1567–77. doi: 10.1002/ece3.4734 (PMC6392356; doi:10.1002/ece3.4734)
Supplement: Supplementary file 1 [file ECE3-9-1567-s001.docx]

**Supplementary material**

**Appendix S1.**

Database reference list

1. Allen LG (2012) Planktonic larval duration, settlement, and growth rates of the young-of-the-year of two sand basses (*Paralabrax nebulifer* and *P. maculofasciatus*: fam. Serranidae) from Southern California. Bulletin of the Southern Californian Academy of Sciences: Vol III: Iss.1.
2. Allen GR & Robertson DR (1997) An annotated checklist of the fishes of Clipperton Atoll, Tropical Eastern Pacific. *Rev. Biol. Trop.* 45:813-843.
3. Allen LG, Pondella DJ & Horn MH (2006) The ecology of marine fishes: California and adjacent waters. University of California Press. London, England. 660p.
4. Bernardi G & Lape J (2005) Tempo and mode of speciation in the Baja California injunct fish species *Anisotremus davidsonii. Mol. Ecol.* 14:4085-4096*.*
5. Böhlke EB, McCosker JE & Böhlke JE (1989) Family Muraenidae. In: Fishes of the western North Atlantic, Böhlke EB (ed.). *Mem. Sears Found. Mar. Res*., Memoir No. 1, 9: 655p.
6. Brothers EB, Williams DM & Sale PF (1983) Length of larval life in twelve families of fishes at 'One Tree Lagoon', Great Barrier Reef, Australia. *Mar. Biol*. 76: 319-324
7. Carr MH & Reed DC (1992) Conceptual issues relevant to marine harvest refuges: examples from temperate reef fishes. *Can. J. Fish. Aquat. Sci.* 50:2019-2028.
8. Chapman MR, Kramer DL (2000) Movements of fishes within and among fringing coral reefs in Barbados. *Environ Biol Fish* 57:11-24.
9. Craig MT, Hastings PA, Pondella II DJ, Robertson DR & Rosales-Casian JA (2006) Phylogeography of the flag cabrilla *Epinephelus labriformis* (Serranidae): implications for the biogeography of the Tropical Eastern Pacific and the early stages of speciation in a marine shore fish. *J. Biogeogr*.33:969-979.
10. Cowen RK (1991) Variation in the planktonic larval duration of the temperate wrasse *Semicossyphus pulcher*. Mar. Ecol. Prog. Ser. 69:9-15.
11. Fischer W, Krupp F, Schneider W, Sommer C, Carpenter KE & Niem VW (1995) Guia FAO para la identificacion de especies para los fines de pesca. Pacifico centro-oriental. Volumen III. Vertebrados - parte 2. Roma, Italy.
12. Floeter SR, Ferreira CEL, Dominici-Arosemena A, Zalmon IR (2004) Latitudinal gradients in Atlantic reef fish communities: trophic structure and spatial use patterns. *J Fish Biol* 64:1680-1699.
13. Froese R, Pauly DP (2011) FishBase. World Wide Web electronic publication. www.fishbase.org. Accessed 2013.
14. Gallardo-Cabello M, Sarabia-Mendez M, Espino-Barr E, Anislado-Tolentino V (2010) Biological aspects of Lutjanus peru in Bufadero Bay, Michoacan, Mexico: growth, reproduction and condition factors. *Rev Biol Mar Oceanogr* 45(2):205-215.
15. Gibran FZ & Castro RMC (1999) Activity, feeding behaviour and diet of *Ogcocephalus verspertilio* in southern West Atlantic. *J. Fish. Biol*. 55:588-595.
16. Gollub AR (1974) Tidal activity rhythms in two species of intertidal clingfish (Gobiesocidae) in the northern Gulf of California. Master Thesis. University Arizona. http://arizona.openrepository.com/arizona/bitstream/10150/566410/1/AZU_TD_BOX276_E9791_1974_305.pdf
17. Grove JS, Lavenberg RJ (1997) The Fishes of Galapagos Islands. Stanford University Press. Stanford, California. 871pp.
18. Hobson ES (1965) Diurnal-Nocturnal Activity of some inshore fishes in the Gulf of California. *Copeia* 3:291-302.
19. Hobson ES (1972) Activity of Hawaiian reef fishes during the evening and morning transitions between daylight and darkness. *Fish Bull* 70(3):715-740.
20. Hobson ES, McFarland WN, Chess JR (1981) Crepuscular and nocturnal activities of Californian nearshore fishes, with consideration of their scotopic visual pigments and the photic environment. *U S Fish Bull* 79:1–30.
21. Hunter JR (1967) Colour changes of prejuvenile goatfish, *Pseudopeneus grandisquamis*, after confinement in a shipboard aquarium. *Copeia* 4:850-852.
22. Johnson GD, Rosenblatt RH (1988) Mechanisms of light organ occlusion in flashlight fishes, family Anomalopidae (Teleostei: Beryciformes), and the evolution of the group. *Zoo J Linn Soc* 94:65-96.
23. Leis JM (1984) Larval fish dispersal and the East Pacific barrier. *Oceanogr. Trop.* 19: 181-192.
24. Luiz OJ, Allen AP, Robertson DR, Floeter SR, Kulbicki M, Vigliola L, Becheler R, Madin JS (2013) Adult and larval traits as determinants of geographic range size among tropical reef fishes. *Proc. Na.t Acad. Sci.* 110(41):16498-16502.
25. Myers MC, Wagner J, Vaughan C (2011) Long-term comparison of the fish community in a Costa Rican rocky marine reserve. *Rev. Biol.* *Trop.* 59(1):233-246
26. Nelson JS (2004) Fishes of the world. Fourth edition. John Wiley & Sons, Inc. Hoboken, New Jersey. 601 pp.
27. Pittman SJ, Monaco ME, Friedlander AM, Legare B, Nemeth RS, Kendall MS, Poti M, Clark RD, Wedding LM & Caldow C (2014) Fish with chips: tracking reef fish movement to evaluate size and connectivity of Caribbean marine protected areas*. Plos one*. 9: e96028.
28. Robertson DR, Allen G (2016) Fishes: East Pacific. An Identification Guide to the Shore-Fish Fauna of the Tropical Eastern Pacific. (Copyright Smithsonian Institution, Left Coast R&C, Santa Cruz, California). http://biogeodb.stri.si.edu/sftep/en/pages, accessed in 2016.
29. Robertson DR, Grove JS & McCosker JE (2004) Tropical transpacific shore fishes. *Pacific Science.* 58: 507-565.
30. Salazar CE (1997) Diurnal behaviours of *Anguilla marmorata* in streams of Moorea, French Polynesia. p37. In: The biology and geomorphology of tropical islands, students research papers, fall 1997.
31. Salinas de Leon P, Rastoin E & Acuña-Marrero D (2015) First record of a spawning aggregation for the tropical eastern Pacific endemic grouper *Mycteroperca olfax* in the Galapagos Marine Reserve. *J. Fish. Biol*. 87: 179-186.
32. Schmitz L, Wainwright P (2011) Nocturnality constrains morphological and functional diversity in the eyes of reef fishes. *BMC Evol Biol* 11:338
33. Shanks AL & Eckert GL (2005) Population persistence of California current fishes and benthic crustaceans: a marine drift paradox. *Ecol. Monogr.* 75: 505-524.
34. Soria G, Torre-Cosio J, Munguia-Vega A, Marinone SG, Lavin MF, Cinti A & Moreno-Baez M (2014) Dynamic connectivity patterns from an insular marine protected area in the Gulf of California. J. Mar. Systems. 129: 248-258.
35. Thomson DA, Findley LT & Kerstitch AN (2000) Reef fishes of the sea of Cortez: the rocky-shore fishes of the Gulf of California. University of Texas Press. Austin, Texas. 353 p.
36. Thresher RE (1984) Reproduction in reef fishes (Neptune City: TFH Publications).
37. Victor BC & Wellington GM (2000) Endemism and the pelagic larval duration of reef fishes in the eastern Pacific Ocean. *Mar. Ecol. Prog. Ser.* 205: 241-248.
38. Victor BC, Wellington GM, Robertson DR & Ruttenberg BI (2001) The effect of the niño-southern oscillation event on the distribution of reef-associated labrid fishes in the eastern Pacific Ocean. *Bull. Mar. Sci*. 69:279-288.

**Appendix S2.**

Validation of model without (Fig. 1a) and with (Fig. 1b) logit transformation


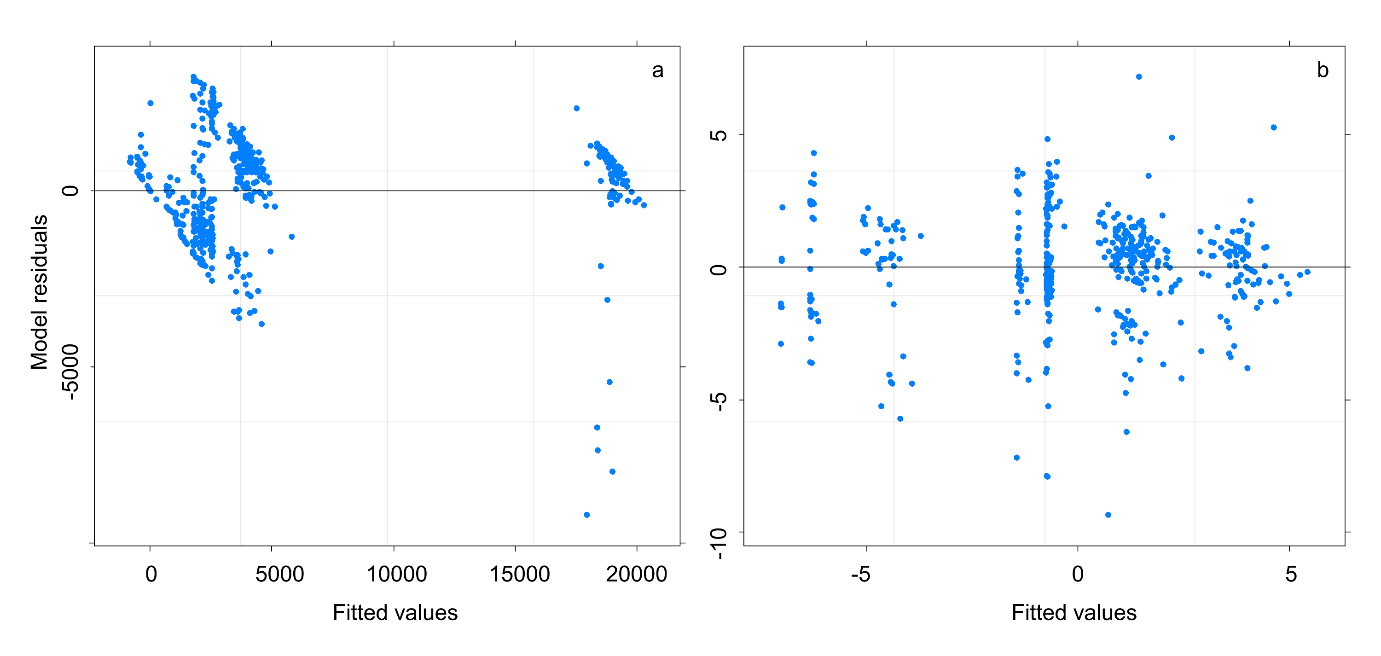


**Fig. 1.** Validation of model assumptions. Plot of model fitted values vs. residuals without (a) and with a logit transformation (b).

**Appendix S3.**

**Table 1.** Summary results of model averaging of the effects of eight traits on range size of reef fishes in the TEP. *Estimates represent standardized effect sizes. Reference levels: low adult mobility, non-pelagic spawners, non-aggregative and diurnal.

| Variable | PLD subset | | | | Complete dataset | | | |
| --- | --- | --- | --- | --- | --- | --- | --- | --- |
|  | Estimate* | SE | z | p | Estimate* | SE | z | p |
| Intercept | -1.22 | 3.62 | 0.33 | 0.74 | -1.08 | 3.05 | 0.35 | 0.72 |
| Medium mobility | 0.88 | 0.56 | 1.55 | 0.12 | **1.42** | **0.31** | **4.59** | **0.00** |
| High mobility | 0.14 | 0.48 | 0.29 | 0.78 | **0.88** | **0.35** | **2.50** | **0.01** |
| Facultative aggregations | 0.19 | 0.41 | 0.46 | 0.65 | 0.01 | 0.13 | 0.10 | 0.92 |
| Aggregations | 0.06 | 0.27 | 0.21 | 0.83 | 0.01 | 0.11 | 0.08 | 0.94 |
| Pelagic spawner | 0.35 | 0.55 | 0.63 | 0.53 | **1.81** | **0.34** | **5.26** | **0.00** |
| PLD | 0.03 | 0.20 | 0.13 | 0.90 |  |  |  |  |
| Crepuscular | -0.09 | 0.49 | 0.18 | 0.85 | -0.67 | 0.70 | 0.96 | 0.34 |
| Nocturnal | -0.20 | 0.38 | 0.52 | 0.60 | **-0.66** | **0.31** | **2.15** | **0.03** |
| Body size | 0.67 | 0.45 | 1.48 | 0.14 | **0.63** | **0.29** | **2.18** | **0.03** |
| Medium mobility x Pel. spawner | -0.14 | 0.51 | 0.27 | 0.79 | **-2.15** | **0.66** | **3.27** | **0.00** |
| High mobility x Pel. spawner | -0.14 | 0.51 | 0.28 | 0.78 | **-1.79** | **0.65** | **2.76** | **0.01** |
